# REPLY TO “The meaningfulness of searching for minimal exposure duration to understand visual perception”

**DOI:** 10.1038/s41467-026-75040-6

**Published:** 2026-07-09

**Authors:** Renzo C. Lanfranco, Pietro Amerio, Andrés Canales-Johnson, Hugh Rabagliati, Axel Cleeremans, David Carmel

**Affiliations:** 1 Department of Clinical Neuroscience, Karolinska Institutet, Stockholm, Sweden; 2 Consciousness, Cognition & Computation Group, Center for Research in Cognition & Neurosciences, ULB Neuroscience Institute, Université libre de Bruxelles, Brussels, Belgium; 3 Neuropsychology and Cognitive Neurosciences Research Center, Faculty of Health Sciences, Universidad Católica del Maule, Talca, Chile; 4 Department of Psychology, University of Cambridge, Cambridge, UK; 5 Neuroscience Center, Helsinki Institute of Life Sciences, University of Helsinki, Helsinki, Finland; 6 Department of Psychology, University of Edinburgh, Edinburgh, UK; 7 School of Psychological Sciences, Victoria University of Wellington, Wellington, New Zealand

**Keywords:** Consciousness, Perception

**replying to** P. Mamassian & M. Wexler. *Nature Communications* 10.1038/s41467-026-75041-5 (2026)

We welcome Mamassian and Wexler’s interest in our study^1^, as well as the opportunity to clarify why we agree with some of their points but reject their conclusion. Mamassian and Wexler posit that due to the visual system’s temporal integration characteristics, the manipulations of stimulus duration that we used are effectively contrast manipulations, and thus our findings, such as the shorter duration required to detect upright compared to inverted faces (a face inversion effect, FIE), may be due to upright faces having a lower contrast threshold. We completely agree that duration manipulations are manipulations of stimulus intensity. We firmly disagree, however, with Mamassian and Wexler’s assertion that this limits the value of using minimal exposure durations to reveal important properties of the visual system. Minimal durations are not only as useful as minimal contrasts for such purposes, but also offer distinct methodological advantages.

Stimulus duration manipulations have been used extensively to investigate diverse visual functions, including scene perception^2,3^, emotion processing^4^, and visual awareness^5,6^. Our recent contribution capitalised on the newly-developed ability to display stimuli briefly enough to obviate the need for masking, enabling characterisation of minimal required exposures without contamination from other stimuli. Under Bloch’s law^7^, an increase in duration corresponds to a proportional increase in perceived contrast. According to Mamassian and Wexler, this means that duration manipulations could be replaced by manipulations of contrast. We agree, but by the same logic the opposite also applies: if contrast and duration are interchangeable, then either of them can be used to measure minimal exposures, and those minimal exposures can be informative about the visual system’s processing priorities.

But are duration and contrast truly interchangeable (i.e., conform to Bloch’s law) for complex stimuli such as faces? This is an empirical question. As we noted (and as Mamassian and Wexler acknowledge), there are valid reasons to question whether this is the case^8,9^. The contrast judgement task Mamassian and Wexler used provides preliminary support for a relationship between faces’ perceived contrast and duration. Crucially, however, their task, in which observers compared images’ overall perceived contrast, is fundamentally different from—and may be entirely independent of—tasks that require extraction of meaningful content from a display. Notably, Mamassian and Wexler’s data do not address their main suggestion—that findings like the FIE could be due to differential contrast thresholds—because they only examined perceived contrast, and furthermore, did so without comparing the perceived contrast of upright and inverted faces and without examining observers’ ability to make judgements about the images’ content. Their findings are therefore orthogonal to our key idea: That differences in sensitivity to the meaning of various stimulus categories cannot be attributed to differences in stimulus energy alone, because it is possible to observe sensitivity differences even when stimulus energy is identical (as it is in upright and inverted faces). To demonstrate this point directly, we replicated the FIE (Fig. 1A) for brief images that have the same perceived, as well as physical, contrast (Fig. 1B). All participants provided informed consent, and the study was approved by the ethics committee of the Faculty of Psychological Science and Education at Université libre de Bruxelles.

**Fig. 1:**
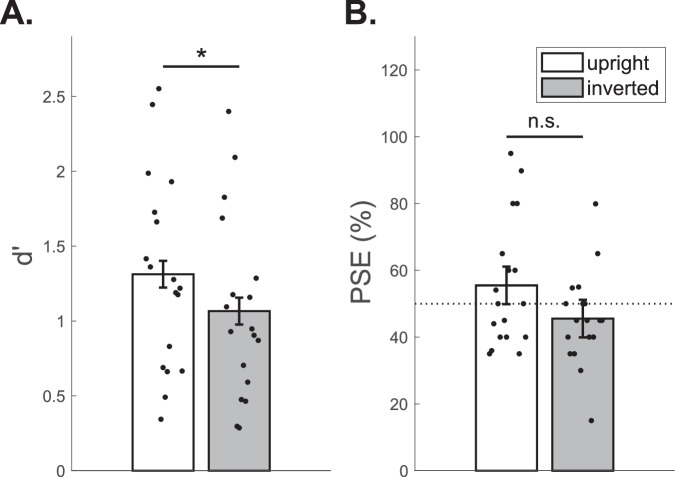
Differential detection of upright and inverted faces with similar physical and perceived contrast. Participants (*n* = 18) gave informed consent and performed two tasks in counterbalanced order—face detection and contrast judgement. On each trial, they were shown a display containing an intact face and a scrambled image to the left and right of fixation (location randomised), as in Experiment 1 of Lanfranco et al. (2024). Display duration was 4.9 ms, midway between the durations showing the largest FIE in that experiment (for full details, see Supplementary Note 1). **A** Face detection. Participants reported the location of the intact face. Results demonstrate a detection advantage for upright over inverted faces (paired t-test, *t*(17) = 2.75, *p* = 0.027; Bayesian t-test, *BF*_10_ = 4.10, 95% credible interval = [0.04, 0.4]), replicating our previous finding. **B** Contrast judgement. In each display, the face’s contrast was varied using a staircase method, scramble contrast was fixed at 50%, and participants reported the side of the higher-contrast stimulus. The points of subjective contrast equality (PSEs) for upright and inverted faces did not differ from the scramble’s physical 50% contrast (indicated by the dashed grey line; one-sample t-tests, upright: *t*(17) = 1.21, p = 0.487, *BF*_10_ = 0.46, 95% credible interval = [44.5, 64.5]%, inverted: *t*(17) = −1.36, p = 0.381, *BF*_10_ = 0.54, 95% credible interval = [37.7, 52.1]%), nor from each other (paired t-test, *t*(17) = 1.76, p = 0.191; *BF*_10_ = 0.88, 95% credible interval = [−2.26, 19.8]). If upright faces had been perceived as having a higher contrast than inverted faces, this could potentially mediate the detection FIE – however, in such a case the PSE would be lower for upright than for inverted faces, the opposite of the numerical trend observed here. See Supplementary Note 2 and Supplementary Fig. 1 for additional analyses comparing the two tasks directly. Taken together, these results demonstrate that the visual system can be differentially attuned to stimuli that are identical in both physical and perceived contrast. Error bars represent ±1 standard error with repeated-measures correction^26^. *p*-values are two-tailed and corrected for multiple comparisons. * *p* < 0.05, n.s.: non-significant.

Contrary to Mamassian and Wexler’s description, we did not aim to determine *absolute* minimal exposure durations. As we noted in our paper’s^1^ ‘Discussion’, the durations we found are not absolute minima: Although they set upper bounds on the minimal exposures required to process various stimulus attributes, shorter durations may yet be found with different stimulus or display characteristics. The critical point, though, is not the durations themselves but the sequence of processing stages that is revealed as minimal exposures increase: detection of stimulation, detection of faces as intact objects, engagement of face-specific processes, and processing of emotional content. It would indeed be interesting to determine whether this sequence is replicated when the minimal exposures are defined by contrast rather than duration, but such a claim requires empirical verification and cannot simply be assumed.

Extending Bloch’s law to complex stimuli such as faces is challenging because contrast is not uniformly distributed across their constituent features. For example, facial contrast tends to be highest around the eyes and mouth, and multiplicative contrast manipulations therefore affect different regions of the stimulus unevenly. This suggests that Bloch’s law may hold for some aspects of perception, such as overall contrast, but not others, such as the meaningful information conveyed by specific features or feature configurations. Consistent with this idea, the few studies that have examined Bloch’s law with complex stimuli report systematic violations. Identification of intricate contour shapes requires more total energy at longer exposure durations^8,9^, and similar deviations appear in detection and visual memory tasks involving both abstract and realistic stimuli, including faces and houses^10^. A key difference between simple and complex stimuli is that the latter contain many heterogeneous features that must be integrated to form a perceptual judgement. These features may vary in informativeness and in the stimulus energy needed for their detection and discrimination. Although individual features may comply with Bloch’s law when considered in isolation, the integration process can introduce inefficiencies that produce departures from Bloch’s law at the level of the whole stimulus^11^.

These considerations also suggest that, when the goal is to determine visual priorities, manipulating stimulus duration may be preferable to manipulating contrast, as this allows the images themselves to remain identical across conditions. Keeping the images constant provides several advantages: Unlike contrast manipulations, duration manipulation eliminates the need to linearise stimulator output (gamma calibration), which is essential to compensate for the fact that all modern displays feature a non-linear relation between luminance settings and outputs^12^. Furthermore, because modern displays use a fixed and discrete set of luminance levels (typically 0–255), reducing contrast can introduce information loss: pixels that originally differed slightly in luminance may be mapped to the same level, eliminating fine visual detail. This loss of information also alters the image’s spatial frequency composition and distribution—which may particularly impact the processing of complex stimuli, where (similar to contrast) spatial frequencies are not uniformly distributed, and where different spatial frequencies convey different perceptual attributes^13–16^. In faces, for example, low spatial frequencies encode global and configural cues important for recognising emotional expressions^17–20^ (e.g., the broad mouth curvature signalling happiness or global patterns around the eyes signalling fear), whereas high spatial frequencies convey fine-grained details critical for identity^13,21^ and gaze direction^22^. Critically, manipulating duration preserves all the information contained in an image while varying the amount of perceptual evidence available to the observer.

Our focus on the extraction of meaning from visual stimuli renders the issue of temporal integration irrelevant in the present context. Temporal integration undoubtedly plays a role in perception, and requires stimulation that exceeds the minimal window for temporal persistence, below which different durations are indistinguishable. But judgements of perceived duration, just like judgements of perceived contrast, do not measure the ability to extract meaningful information from stimuli. Our findings show that detection performance can range from chance-level to near-perfect within a set of exposure durations that are shorter than the minimal persistence window, demonstrating that meaning extraction is independent of temporal persistence.

Similarly, while flicker fusion frequencies provide insight into the visual system’s ability to distinguish temporally distinct visual events, this too is orthogonal to the ability to extract meaningful information from single displays. Notably, flicker fusion thresholds are influenced by the availability of attention^23^, whereas the ability to extract sophisticated aspects of meaning from complex images is unaffected by such availability^24,25^. This suggests that the processes involved in distinguishing or integrating temporally distinct stimuli do not fully overlap with those involved in extracting meaning from visual stimulation. Likewise, we accept Mamassian and Wexler’s point that the duration of stimulus availability in iconic memory may be independent of the duration for which the stimulus was presented, but note that this observation relates to post-perceptual processes—our findings suggest that the type and quantity of content that enters iconic memory in the first place may be strongly influenced by stimulus duration.

To reiterate, our main point is not that stimulus energy is irrelevant to the extraction of meaning from visual stimuli, nor that minimal durations are unrelated to perceived contrast: Minimal exposure means, by definition, a minimal amount of stimulation energy, and this energy can be defined by its duration or by its contrast. However, if different stimulus categories require different minimal exposures independently of their physical energy, as we have shown, this indicates that the visual system is attuned to features that are not determined purely in terms of energy. Furthermore, from a methodological perspective, manipulating duration (unlike manipulating contrast) has the advantage of allowing all other features of the image to be kept constant. This makes the measurement of minimal durations particularly valuable for characterising the visual system’s processing priorities.

## Reporting summary

Further information on research design is available in the Nature Portfolio Reporting Summary linked to this article.

## Supplementary information

Supplementary Information

Reporting Summary

## Data Availability

The data generated in this study have been deposited in the Open Science Framework (OSF) and they are open access (10.17605/OSF.IO/VZST9).

## Code availability

The code generated in this study has been deposited in the Open Science Framework (OSF) and they it is open access (10.17605/OSF.IO/VZST9).
