# Supplementary material for: REPLY TO “The meaningfulness of searching for minimal exposure duration to understand visual perception”
Source: Nat Commun. 2026 Jul 9;17:5977. doi: 10.1038/s41467-026-75040-6 (PMC13351077; doi:10.1038/s41467-026-75040-6)
Supplement: Supplementary file 1 — Supplementary Information [file 41467_2026_75040_MOESM1_ESM.pdf]

Supplementary Information

Minimal exposure durations are meaningful for understanding visual perception

Renzo C. Lanfranco, Pietro Amerio, Andres Canales-Johnson, Hugh Rabagliati, Axel Cleeremans, David Carmel

Supplementary Note 1: Task design and procedure

Eighteen participants (M_{age} = 19.7, range = [18-26]; 16 female; 17 right-handed) performed the face detection and contrast judgment tasks in counterbalanced order (200 trials each, equally distributed between conditions). Stimuli were the same as in Experiment 1 of Lanfranco et al (2024)[1] and were presented on the same custom-made tachistoscope. On each trial of both tasks, participants were shown a display containing an intact face and a scrambled image to the left and right of fixation (location randomised). In both tasks, the intact face could be either upright or inverted (counterbalanced).

In the face detection task, display duration was 4.9 ms, midway between the durations showing the largest FIE in Lanfranco et al.'s Experiment 1. To enable comparison with the second task, stimuli were shown at 50% contrast. Participants reported the side of the intact face.

In the contrast judgment task, the faces' contrast was varied using two PSI-marginal staircases[2] (one for upright and one for inverted faces; 5% steps), while scramble contrast was fixed at 50%. Participants reported the side of the higher-contrast stimulus. Contrast was manipulated by multiplying all pixel values by a contrast scaling factor, after subtracting the background colour (RGB 220), which was added again at the end.

The PSI-marginal staircases allowed measurement of the intact-face contrast at which the face and the scramble had an equal probability of being judged as the higher-contrast stimulus (i.e., point of subjective equality; PSE). Crucially, the PSI-marginal staircase works by updating, after each trial, a shared probability distribution of the values of all parameters of a psychometric function (here, defined as cumulative normal). PSEs were defined as the maximum *a posteriori* values relative to the threshold parameter (slopes, lapse rates and guess rates were treated as nuisance parameters).

Participants performed a short practice prior to each task (32 trials), to familiarise them with the instructions and response schemes. The entire experimental session lasted approximately 80 minutes.

Our sample size was supported by power analysis conducted using the pwr package in R[3], which determined that, for the FIE with 5.3 ms of exposure (Cohen's d = 0.915 based on our previous findings[1]), a paired-sample t-test with an alpha level of 0.05 and a power of 0.95 required 18 participants.

The experiments were approved by the Université libre de Bruxelles Faculty of Psychological Science and Education ethics committee

Supplementary Note 2: Additional analyses

As noted in the main text, we found a detection advantage for upright over inverted faces (an FIE), replicating our previous finding (**Fig. 1A**). This contrasts with the finding that the PSEs for upright and inverted faces did not differ from the scramble's physical 50% contrast, nor from each other (**Fig. 1B)**. Here, we further examine whether location sensitivity (d') and PSE may have nonetheless reflected the same underlying mechanism. To this end, we performed two additional analyses.

First, we standardised both measures (z-scores) and analysed them using a 2 × 2 within-subjects ANOVA with task and face orientation as factors. This analysis showed a significant main effect of face orientation, $F_{(1,17)} = 6.06$, $p = .025$, $\eta_p^2 = .263$, whereas neither the main effect of task ($F_{(1,17)} < 0.01$, $p > .999$), nor the interaction between task and orientation reached significance ($F_{(1,17)} = 0.34$, $p = .565$). Crucially, the effect of orientation was consistent across both measures, with lower values for inverted faces. This is important, because if inverted faces were simply harder to detect because they generate lower perceived contrast —as suggested by Mamassian & Wexler—they should instead have yielded higher PSEs (and higher Z-scores). The lower PSE for inverted faces therefore suggest that perceived contrast alone cannot explain the effect of orientation on information extraction from complex face stimuli.

Second, we examined the correlation between the effects of face inversion on location sensitivity (d') and PSE. If the FIE were accounted for by higher perceived contrast for upright than for inverted faces, there should be a negative correlation between the magnitude of the FIE (Δd' = d' upright minus d' inverted) and the difference of PSEs (ΔPSE = PSE upright minus PSE inverted). The correlation, however, was not significant ($r(16) = .30$, $p = .22$; $\rho(16) = .24$, $p = .33$; see **Supplementary Figure 1**) – and furthermore, the numerical trend was positive—the opposite direction from that predicted by an effective-contrast account, indicating that variability in detection sensitivity cannot be explained by variability in perceived contrast.

In summary, the two additional analyses above indicate that the detection FIE is not explained by differences in perceived or physical contrast.

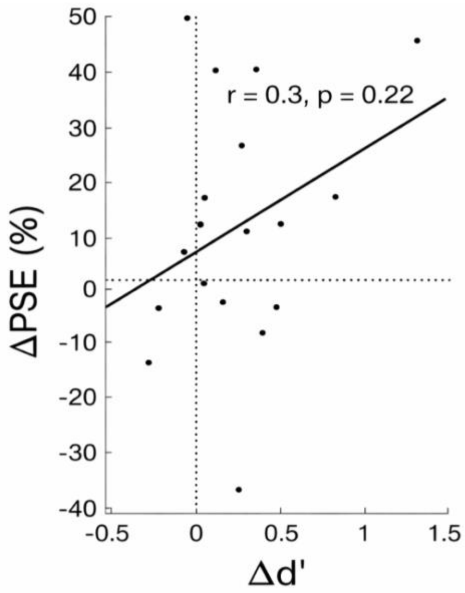

Supplementary Fig. 1. Relationship between detection sensitivity and perceived contrast. Across participants, individual differences in the detection advantage for upright over inverted faces (Δd') did not significantly correlate with corresponding differences in perceived contrast (ΔPSE). The numerical trend was in the opposite direction to the negative correlation that would be expected if PSE differences had accounted for the FIE.

References

1. Lanfranco, R. C., Canales-Johnson, A., Rabagliati, H., Cleeremans, A. & Carmel, D. Minimal exposure durations reveal visual processing priorities for different stimulus attributes. *Nat. Commun.* **15**, 8523 (2024).
2. Prins, N. The psi-marginal adaptive method: How to give nuisance parameters the attention they deserve (no more, no less). *J. Vis.* **13**, 3 (2013).
3. Champely, S. *et al.* pwr: Basic Functions for Power Analysis. (2020).
