# Supplementary material for: REPLY TO “The meaningfulness of searching for minimal exposure duration to understand visual perception”
Source: Nat Commun. 2026 Jul 9;17:5977. doi: 10.1038/s41467-026-75040-6 (PMC13351077; doi:10.1038/s41467-026-75040-6)
Supplement: Supplementary file 2 — Reporting Summary [file 41467_2026_75040_MOESM2_ESM.pdf]

nature portfolio

Corresponding author(s): Renzo Lanfranco

Last updated by author(s): Mar 18, 2026

Reporting Summary

Nature Portfolio wishes to improve the reproducibility of the work that we publish. This form provides structure for consistency and transparency in reporting. For further information on Nature Portfolio policies, see our Editorial Policies and the Editorial Policy Checklist.

Statistics

For all statistical analyses, confirm that the following items are present in the figure legend, table legend, main text, or Methods section.

n/a	Confirmed	
☐	☒	The exact sample size (*n*) for each experimental group/condition, given as a discrete number and unit of measurement
☐	☒	A statement on whether measurements were taken from distinct samples or whether the same sample was measured repeatedly
☐	☒	The statistical test(s) used AND whether they are one- or two-sided<br>*Only common tests should be described solely by name; describe more complex techniques in the Methods section.*
☒	☐	A description of all covariates tested
☐	☒	A description of any assumptions or corrections, such as tests of normality and adjustment for multiple comparisons
☐	☒	A full description of the statistical parameters including central tendency (e.g. means) or other basic estimates (e.g. regression coefficient) AND variation (e.g. standard deviation) or associated estimates of uncertainty (e.g. confidence intervals)
☐	☒	For null hypothesis testing, the test statistic (e.g. *F*, *t*, *r*) with confidence intervals, effect sizes, degrees of freedom and *P* value noted<br>*Give P values as exact values whenever suitable.*
☐	☒	For Bayesian analysis, information on the choice of priors and Markov chain Monte Carlo settings
☒	☐	For hierarchical and complex designs, identification of the appropriate level for tests and full reporting of outcomes
☐	☒	Estimates of effect sizes (e.g. Cohen's *d*, Pearson's *r*), indicating how they were calculated

Our web collection on statistics for biologists contains articles on many of the points above.

Software and code

Policy information about availability of computer code

Data collection	MATLAB 2024b, Psychtoolbox 3
Data analysis	MATLAB 2024b, .R Studio 2025.09.

For manuscripts utilizing custom algorithms or software that are central to the research but not yet described in published literature, software must be made available to editors and reviewers. We strongly encourage code deposition in a community repository (e.g. GitHub). See the Nature Portfolio guidelines for submitting code & software for further information.

Data

Policy information about availability of data

All manuscripts must include a data availability statement. This statement should provide the following information, where applicable:

- Accession codes, unique identifiers, or web links for publicly available datasets
- A description of any restrictions on data availability
- For clinical datasets or third party data, please ensure that the statement adheres to our policy

Data and code have been made available at https://doi.org/10.17605/OSF.IO/VZST9.

Human research participants

Policy information about studies involving human research participants and Sex and Gender in Research.

Reporting on sex and gender	All participants reported that their sex and gender matched. However, sex and gender were irrelevant in our study, so we did not try to have a gender-balanced sample. In our study, we analysed data from 16 female and 2 male participants. Consent was obtained to collect this information, but not to share individual-level data.<br>All our experiments involved visual presentations of face stimuli, which were gender-balanced according to the stimulus set's norms. Per condition, 50% of the face stimuli were female and 50% were male.<br>No sex- or gender-based analyses were performed because they were not relevant to address the project's questions and hypotheses.
Population characteristics	See above
Recruitment	Recruitment through university social media channels and online recruitment systems. It is not clear how self-selection biases (if any) would impact the results.
Ethics oversight	Université Libre de Bruxelles Ethics Committee

Note that full information on the approval of the study protocol must also be provided in the manuscript.

Field-specific reporting

Please select the one below that is the best fit for your research. If you are not sure, read the appropriate sections before making your selection.

☐ Life sciences ☒ Behavioural & social sciences ☐ Ecological, evolutionary & environmental sciences

For a reference copy of the document with all sections, see nature.com/documents/nr-reporting-summary-flat.pdf

Behavioural & social sciences study design

All studies must disclose on these points even when the disclosure is negative.

Study description	Cognitive neuroscience, experimental psychology, quantitative methods
Research sample	Mostly Université Libre de Bruxelles students both attending undergraduate and postgraduate programmes. These samples are in keeping with general research practices in the field but not representative for the general population. Importantly, demographic factors such as gender and education are not hypothesised to impact the findings.
Sampling strategy	Convenience sampling. A retrospective power analysis to capture a small- to medium-size effect (i.e., partial eta-squared of 0.04) between conditions in a repeated-measures ANOVA model with an alpha of 0.05, aiming at a statistical power of 95%, determined that a sample of 19 participants should be sufficient.
Data collection	The experiments were computer based - participants had to sit in front of the LCD tachistoscope screen and respond to a programmed perceptual task. The experimenter was blind to the specific conditions and to the participant's responses.
Timing	Data collection of the experiment took place in 2025, between June and October.
Data exclusions	No participant was excluded.
Non-participation	No participant dropped out.
Randomization	All independent variables were within-subject factors using a repeated-measures design.

Reporting for specific materials, systems and methods

We require information from authors about some types of materials, experimental systems and methods used in many studies. Here, indicate whether each material, system or method listed is relevant to your study. If you are not sure if a list item applies to your research, read the appropriate section before selecting a response.

Materials & experimental systems

n/a	Involved in the study
☒	☐ Antibodies
☒	☐ Eukaryotic cell lines
☒	☐ Palaeontology and archaeology
☒	☐ Animals and other organisms
☒	☐ Clinical data
☒	☐ Dual use research of concern

Methods

n/a	Involved in the study
☒	☐ ChIP-seq
☒	☐ Flow cytometry
☒	☐ MRI-based neuroimaging
